# Prognostic value of lymphovascular invasion in stage II colorectal cancer patients with an inadequate examination of lymph nodes

**DOI:** 10.1186/s12957-021-02224-3

**Published:** 2021-04-18

**Authors:** Zhenyan Gao, Huihua Cao, Xiang Xu, Qing Wang, Yugang Wu, Qicheng Lu

**Affiliations:** 1 Department of General Surgery, The Third Affiliated Hospital of Soochow University and The First People’s Hospital of Changzhou, 185 Juqian Street, Changzhou, 213000 Jiangsu China; 2 Department of General Surgery, Traditional Chinese Medicine Hospital of Kunshan, Suzhou, 215000 Jiangsu China

**Keywords:** Lymphovascular invasion, Stage II colorectal cancer, Adjuvant chemotherapy, Survival, Prognostic factors

## Abstract

**Background:**

Lymphovascular invasion (LVI) is defined as the presence of cancer cells in lymphatics or blood vessels. This study aimed to evaluate the prognostic value of LVI in stage II colorectal cancer (CRC) patients with inadequate examination of lymph nodes (ELNs) and further combined LVI with the TNM staging system to determine the predictive efficacy for CRC prognosis. Adjuvant chemotherapy (ACT) was then evaluated for stage II CRC patients with LVI positivity (LVI+).

**Methods:**

In order to avoid the effects of different ACT regimens, among 409 stage II patients, we chose 121 patients who received FOLFOX regimen and the 144 patients who did not receive ACT as the object of study. LVI was examined by hematoxylin-eosin (HE) staining. Kaplan-Meier analysis followed by a log-rank test was used to analyze survival rates. Univariate and multivariate analyses were performed using a Cox proportional hazards model. Harrell’s concordance index (C-index) was used to evaluate the accuracy of different systems in predicting prognosis.

**Results:**

The LVI+ status was significantly associated with pT stage, degree of differentiation, tumor stage, serum CEA and CA19-9 levels, perineural invasion (PNI), tumor budding (TB), and *KRAS* status. The 5-year overall survival (OS) rate of stage II patients with < 12 ELNs and LVI+ was less than stage IIIA. Multivariate analyses showed that LVI, pT-stage, serum CEA and CA19-9 levels, PNI, TB, and *KRAS* status were significant prognostic factors for stage II patients with < 12 ELNs. The 8th TNM staging system combined with LVI showed a higher C-index than the 8th TNM staging system alone (C-index, 0.895 vs. 0.833). Among patients with LVI+, the ACT group had a significantly higher 5-year OS and 5-year disease-free survival (DFS) than the surgery alone (SA) group (5-year OS, 66.7% vs*.* 40.9%, *P* = 0.004; 5-year DFS, 64.1% vs*.* 36.3%, *P* = 0.002).

**Conclusions:**

LVI is an independent prognostic risk factor for stage II CRC patients. Combining LVI with the 8th TNM staging system improved the predictive accuracy for CRC prognosis. ACT in stage II CRC patients with LVI+ is beneficial for survival.

## Introduction

Colorectal cancer (CRC) is the third most common malignancy and the fourth leading cause of tumor-related deaths worldwide [1]. Although advances have been achieved in early detection and effective treatment, the survival rate of CRC is still poor [2]. Among all CRC patients, approximately one-third are diagnosed as stage II [3]. The National Comprehensive Cancer Network (NCCN) guidelines recommend adjuvant chemotherapy (ACT) for stage III and IV CRC [4]. For stage II CRC, the current guidelines recommend that ACT should be considered for patients at high risk for recurrence [5].

In addition, the current guidelines recommend that at least 12 lymph nodes (LNs) should be examined for nodal evolution [6]. Adequate LN retrieval from the specimen is essential to ensure accuracy in nodal staging [7]. An inadequate examination of lymph nodes (ELNs) may cause a false-negative result or a lower pN stage [8].

**Fig. 1:**
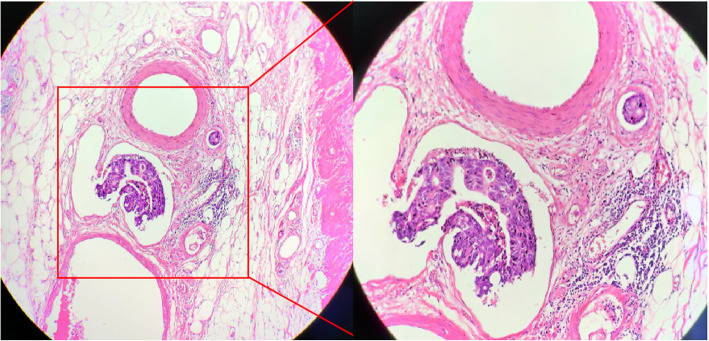
An example of positive lymphovascular invasion (LVI), diagnosed by at least two experienced pathologists on H&E examination. H&E (×100, ×200)

Lymphovascular invasion (LVI) is defined as the presence of cancer cells in lymphatics or blood vessels and is considered to be an early step in lymph node metastasis (Fig. 1) [1]. Many studies have reported that LVI positivity (LVI+) is a critical prognostic indicator in some cancers, including breast, bladder, and gastric cancers [9–11]. It has been reported that the presence of LVI in CRC varies from 4.1 to 89.5% [12]. Currently, few studies have focused on LVI in stage II CRC with inadequate ELNs. Moreover, no study involving the combination of LVI and the TNM staging system in CRC patients has been published.

**Fig. 2:**
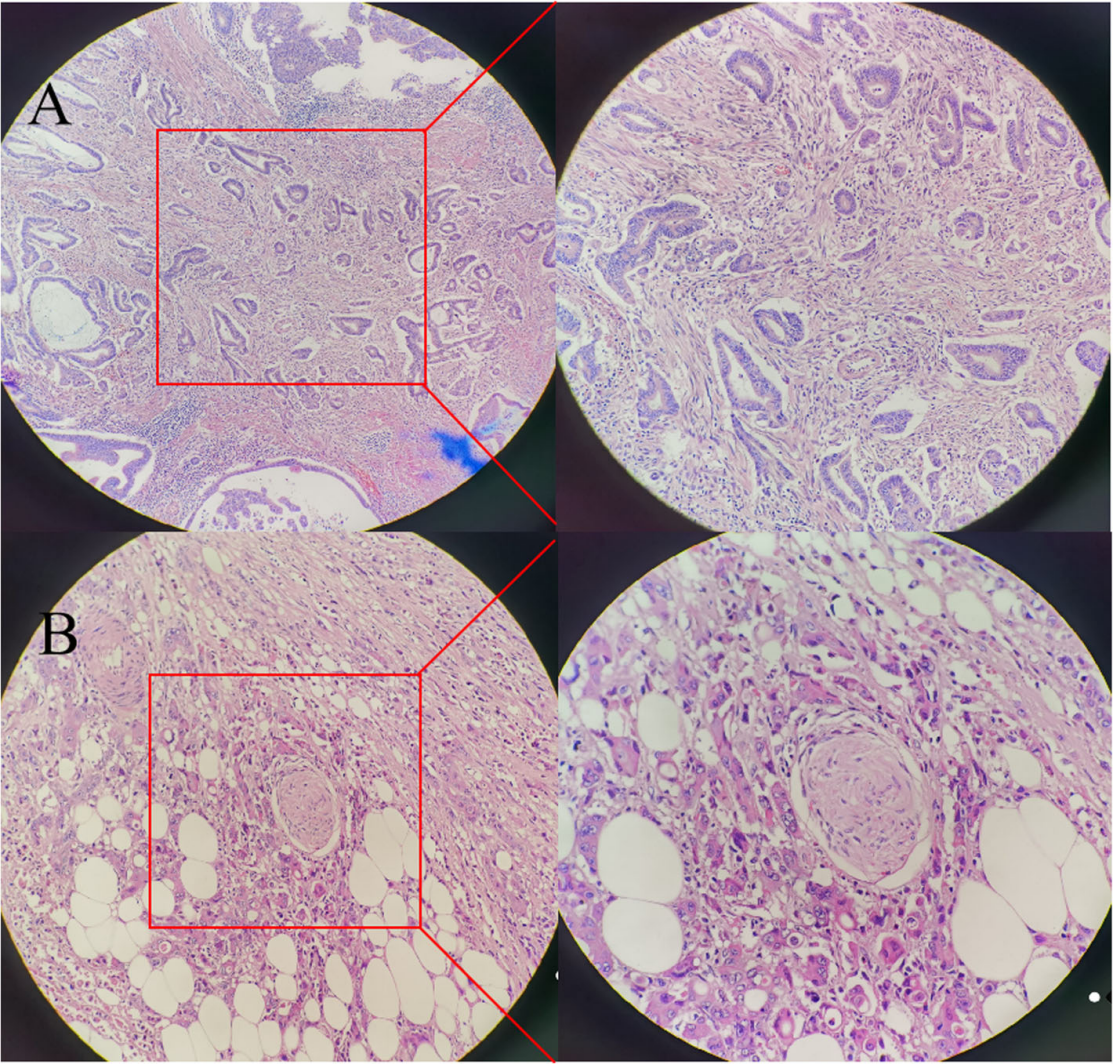
Example of tumor budding (TB), H&E (×200, ×400) (**a**); an example of positive perineural invasion (PNI), H&E (×200, ×400) (**b**)

Tumor budding (TB) was defined as a single tumor cell or cluster comprising less than five cells at the invasive front [13]. At the International Tumor Budding Consensus Conference (ITBCC), it was clearly stated that tumor budding is an independent prognostic factor for CRC. The reason why tumor budding has not yet developed into routine clinical practice is because there is no consensus on the scoring method. This study used the methods proposed by ITBCC in clinical practice and studied the relationship between TB and the survival of patients with stage II CRC with ELNs. Cutoffs used were the following: low, 0–9 buds; and high, ≥ 10 buds (Fig. 2a) [13]. Perineural invasion (PNI) is the process of nerve tumor infiltration, including tumor cells located in the three layers of the peripheral nerve sheath or adjacent to the nerve, and involving at least one third of its surroundings [14, 15]. PNI has become a key pathological feature of many malignant tumors, including malignant tumors of the stomach, colon and rectum, pancreas, and biliary tract (Fig. 2b) [16–19]. At present, there is no consensus regarding the inclusion of PNI in staging, although PNI has been proven to be a sign of poor survival in colorectal cancer.

We conducted the current study to evaluate the prognostic value of LVI in stage II CRC patients with inadequate ELNs and combined LVI with the tumor-node-metastasis (TNM) staging system to determine the predictive efficacy for CRC prognosis. ACT was then evaluated for stage II CRC patients with LVI+.

## Materials and methods

### Patients

We retrospectively examined the clinicopathologic records of CRC patients who were treated at the Third Affiliated Hospital of Soochow University between February 2007 and February 2013. The inclusion criteria were as follows: adenocarcinoma confirmed by histopathology, curative resection with lymphadenectomy, no neoadjuvant chemoradiation, complete clinicopathologic records, absence of distant metastases. The exclusion criteria were as follows: received neoadjuvant chemoradiation, incomplete clinicopathologic records, lost to follow-up, distant metastases. CRC stage was classified according to the 8th edition of the American Joint Committee on Cancer (AJCC) TNM staging system. Follow-up was carried out by telephone calls, emails, and on-site visits. Informed written consent was obtained from all CRC patients. The study was approved by the Ethics Committee of the Third Affiliated Hospital of Soochow University.

### ACT regimens for stage II CRC patients

After curative resection, some stage II CRC patients chose to receive ACT for further treatment. The ACT regimen was established by our clinicians based on the patient’s general performance, clinicopathologic features, and operative factors. A 6-month oxaliplatin-based regimen (FOLFOX [5-fluorouracil with oxaliplatin] or CapeOx [capecitabine with oxaliplatin]) was recommended for stage II CRC patients. For those patients with a contraindication to oxaliplatin, a 6-month fluoropyrimidine-based regimen (5-FU/LV [5-fluorouracil/leucovorin] or 5-FU [5-fluorouracil]) was an acceptable alternative.

### Data collection and LVI examination

Patient medical records were reviewed to obtain clinicopathologic data. Age, sex, tumor size, tumor location, LVI, TNM stage, degree of differentiation, ELNs, serum CEA and CA19-9 levels, perineural invasion, tumor budding, and *KRAS* status were recorded. Specimens were fixed in formalin, then cut into multiple slices. The histopathological examination was performed using a 5-mm-thick longitudinal whole tissue section. Slices were then embedded in paraffin and stained with hematoxylin-eosin (HE). All H&E slides were evaluated by at least two experienced pathologists, who independently assessed LVI, TB, and PNI. Lymphovascular invasion (LVI) is defined as the presence of cancer cells in lymphatics or blood vessels [1]. Tumor budding (TB) was defined as a single tumor cell or cluster comprising less than five cells at the invasive front [13]. Perineural invasion (PNI) is the process of nerve tumor infiltration, including tumor cells located in the three layers of the peripheral nerve sheath or adjacent to the nerve, and involving at least one third of its surroundings [14, 15]. The Amplified Refractory Mutation System (ARMS) was used to detect *KRAS* mutations in colorectal cancer.

### Statistical analysis

All analyses were performed using the SPSS (version 25.0 software; IBM, Chicago, IL, USA) and R software (version 3.0.0; www.r-project.org). Statistical significance was tested using Student’s *t*-test and chi-squared test. Univariate and multivariate analyses were performed using a Cox proportional hazards model. Kaplan-Meier analysis followed by a log-rank test was used to analyze survival rates. Harrell’s concordance index (C-index) was used to evaluate the accuracy of different systems in predicting prognosis. All statistical analyses were two-sided, and a *P <* 0.05 was considered statistically significant.

## Results

### Patient characteristics

One thousand four hundred and twenty CRC patients from February 2007 to February 2013 who met the inclusion criteria were evaluated in this study. The clinicopathologic characteristics of 1420 CRC patients are listed in Table 1. Of all CRC patients, there were 822 males (57.9%) and 598 females (42.1%); 47.1% of patients were ≤ 65 years of age. and 52.9% of patients were > 65 years of age. The mean number of tumor size was 4.31 ± 2.38 for LVI+ group and 3.98 ± 2.09 for LVI− group. The number of stages I, II, and III patients were 173, 409, and 838, respectively. Of the 409 stage II patients, 144 patients did not receive ACT, 121 patients received FOLFOX regimen, 56 patients received CapeOx regimen, 48 patients received 5-FU/LV regimen, and 40 received 5-FU regimen.

**Table 1:** Clinicopathologic characteristics of CRC patients. One thousand four hundred and twenty CRC patients from February 2007 to February 2013 who met the inclusion criteria were analyzed in this study. The clinicopathologic characteristics of 1420 CRC patients and the 409 stage II patients are listed in Table 1. Of the 409 stage II patients, 145 patients (35.5%) were LVI+ and 264 (64.5%) were LVI−

Parameters	Overall patients	Stage II patients
	*n* (%)	*n* (%)
Sex		
Female	598 (42.1)	169 (41.3)
Male	822 (57.9)	240 (58.7)
Age (years)		
≤65	669 (47.1)	198 (48.4)
>65	751 (52.9)	211 (51.6)
Tumor site		
Colon	846 (59.6)	217 (53.1)
Rectum	574 (40.4)	192 (46.9)
Tumor size (cm)		
≤4	688 (48.5)	209 (51.1)
>4	732 (51.5)	200 (48.9)
Lymphovascular invasion		
Positive	486 (34.2)	145 (35.5)
Negative	934 (65.8)	264 (64.5)
T-stage		
T1	233 (16.4)	
T2	393 (27.7)	
T3	457 (32.2)	218 (53.3)
T4	337 (23.7)	191 (46.7)
N-stage		
N0	582 (41.0)	409 (100.0)
N1	498 (35.1)	
N2	340 (23.9)	
Differentiation degree		
Well	666 (46.9)	198 (48.4)
Moderate	582 (41.0)	182 (44.5)
Poor	172 (12.1)	29 (7.1)
CEA		
≤5ng/ml	836 (58.9)	243 (59.4)
>5ng/ml	584 (41.1)	166 (40.6)
CA19-9		
≤37U/ml	1106 (77.9)	322 (78.7)
>37U/ml	314 (22.1)	87 (21.3)
Retrieved LN		
<12	564 (39.7)	164 (40.1)
≥12	856 (60.3)	245 (59.9)
Treatment		
ACT	960 (67.6)	265 (64.8)
SA	460 (32.4)	144 (35.2)
TNM stage		
I	173 (12.2)	
II	409 (28.8)	
IIA	174 (12.3)	
IIB	121 (8.5)	
IIC	114 (8.0)	
III	838 (59.0)	
IIIA	324 (22.8)	
IIIB	287 (20.2)	
IIIC	227 (16.0)	
KRAS status		
Wild type	975 (68.7)	248 (60.6)
Mutant type	445 (31.3)	161 (39.4)
PNI		
Positive	276 (19.4)	90 (22.0)
Negative	1144 (80.6)	319 (78.0)
TB		
Low	995 (70.1)	294 (71.9)
High	425 (29.9)	115 (28.1)

*CRC* Colorectal cancer; *LN* Lymph nodes; *PNI* Perineural invasion; *TB* Tumor budding

In order to avoid the effects of different ACT regimens, among 409 stage II patients, we chose 121 patients who received FOLFOX regimen and the 144 patients who did not receive ACT as the object of study. Finally, these 265 stage II patients were analyzed in this study whose clinicopathologic characteristics are listed in Table 1. One hundred patients (37.7%) were LVI+ and 165 (62.3%) were LVI−. We divided these stage II patients into ACT and surgery alone (SA) groups.

### Occurrence of LVI in stage II CRC patients

As shown in Table 2, the incidence of LVI in 265 stage II CRC patients is listed based on clinicopathologic characteristics. The LVI status was significantly associated with pT stage, degree of differentiation, tumor stage, serum CEA and CA19-9 levels, perineural invasion, tumor budding, and *KRAS* status. The incidence of LVI in pT3, pT4a, and pT4b stages was 22.9%, 47.7%, and 64.3%, respectively. There was a statistically significant difference between LVI and pT stage. No significant difference existed with respect to sex, age, tumor size, tumor site, and ELNs.

**Table 2:** Occurrence of LVI in 265 stage II CRC patients. The incidence of LVI in stage II CRC patients is listed in Table 2 according to clinicopathologic characteristics. The LVI status was significantly associated with pT stage, degree of differentiation, tumor stage, serum CEA and CA19-9 levels, perineural invasion, tumor budding, and *KRAS* status. No significance existed in sex, age, tumor size, tumor site, and ELNs

Parameters	LVI (+)	LVI (−)	*P* value	LVI (+) rate (%)
	*n*	*n*		
Sex			0.563	
Female	43	65		43/108 (39.8%)
Male	57	100		57/157 (36.3%)
Age (years)			0.102	
≤65	54	72		54/126 (42.9%)
>65	46	93		46/139 (33.1%)
Tumor site			0.276	
Colon	59	86		59/145 (40.7%)
Rectum	41	79		41/120 (34.2%)
T-stage			< 0.001	
T3	33	111		33/144 (22.9%)
T4a	31	34		31/65 (47.7%)
T4b	36	20		36/56 (64.3%)
Tumor size (cm)			0.186	
≤4	48	93		48/141 (34.0%)
>4	52	72		52/124 (41.9%)
Differentiation degree			< 0.001	
Well	38	97		38/135 (28.1%)
Moderate	52	66		52/118 (44.1%)
Poor	10	2		10/12 (83.3%)
CEA			< 0.001	
≤5ng/ml	28	133		28/161 (17.4%)
>5ng/ml	72	32		72/104 (69.2%)
CA19-9			< 0.001	
≤37U/ml	61	151		61/212 (28.8%)
>37U/ml	39	14		39/53 (73.6%)
Retrieved LN			0.062	
<12	48	60		48/108 (44.4%)
≥12	52	105		52/157 (33.1%)
Treatment			< 0.001	
ACT	78	43		78/101 (64.5%)
SA	22	122		22/144 (15.3%)
II			0.023	
IIA	37	76		37/113 (32.7%)
IIB	34	44		34/78 (43.6%)
IIC	39	35		39/74 (52.7%)
KRAS status			< 0.001	
Wild type	39	121		39/160 (24.4%)
Mutant type	61	44		61/105 (58.1%)
PNI			< 0.001	
Negative	66	141		66/207 (31.9%)
Positive	34	24		34/58 (58.6%)
TB			< 0.001	
Low	59	136		59/195 (30.3%)
High	41	29		41/70 (58.6%)

*CRC* Colorectal cancer; *LN* Lymph nodes; *LVI* Lymphovascular invasion; *PNI* Perineural invasion; *TB* Tumor budding

Table 2. Occurrence of LVI in 265 stage II CRC patients

### Overall survival of CRC patients

**Fig. 3:**
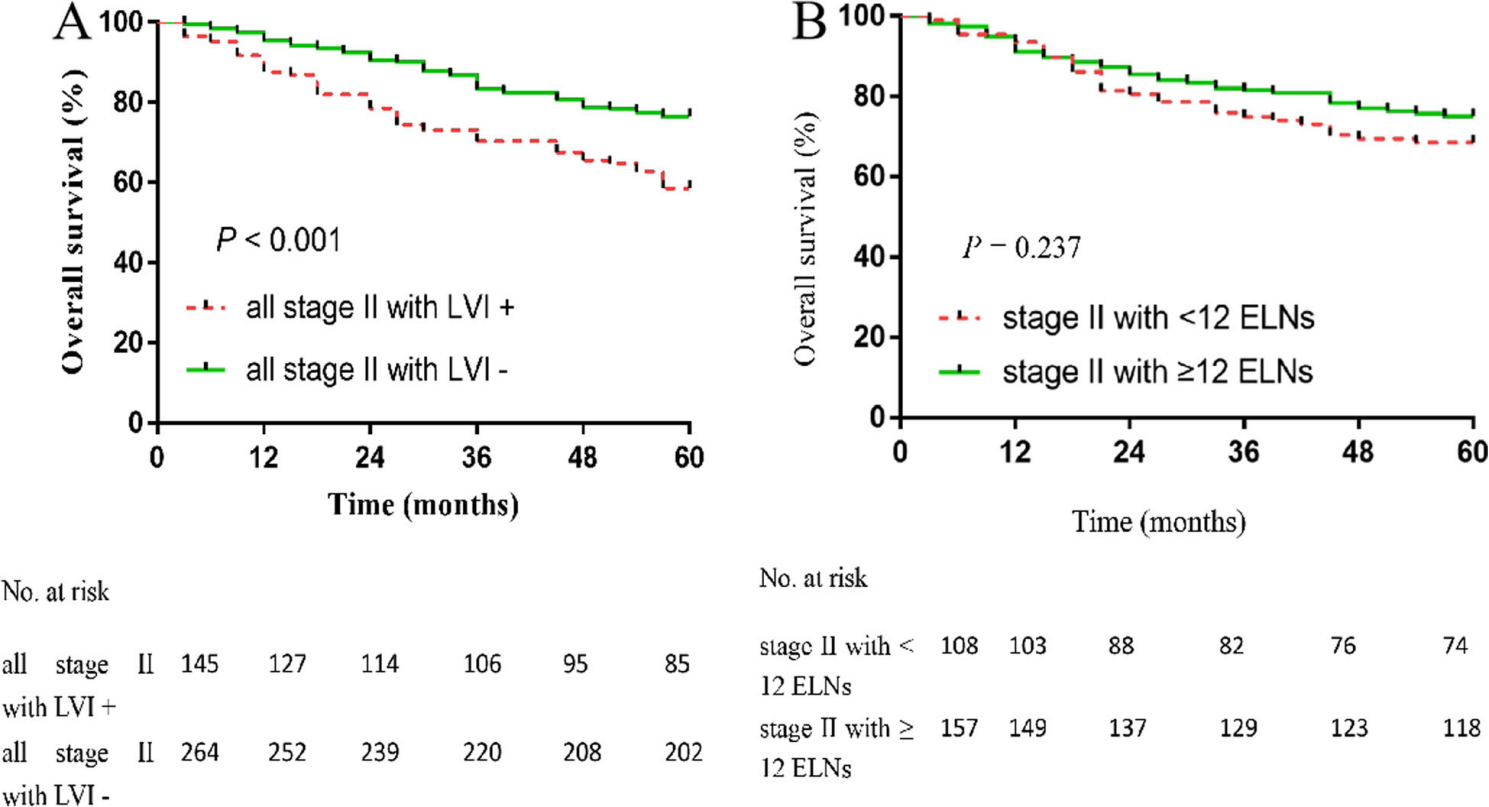
The 5-year overall survival (OS) rate among all stage II CRC patients according to lymphovascular invasion (LVI). We found that in all stage II patients, the 5-year OS rate of the LVI− group was greater than the LVI+ group (76.5% vs. 58.6%; *P* < 0.001, **a**). The 5-year overall survival (OS) rate among stage II CRC patients according to examined lymph nodes (ELNs). We divided the stage II patients into ELNs < 12 and ELNs ≥ 12 groups. The 5-year OS rate of the ELNs ≥ 12 group (75.2%) was greater than the ELNs < 12 group (68.5%); however, there was no statistical significance (*P* = 0.237, **b**)

**Fig. 4:**
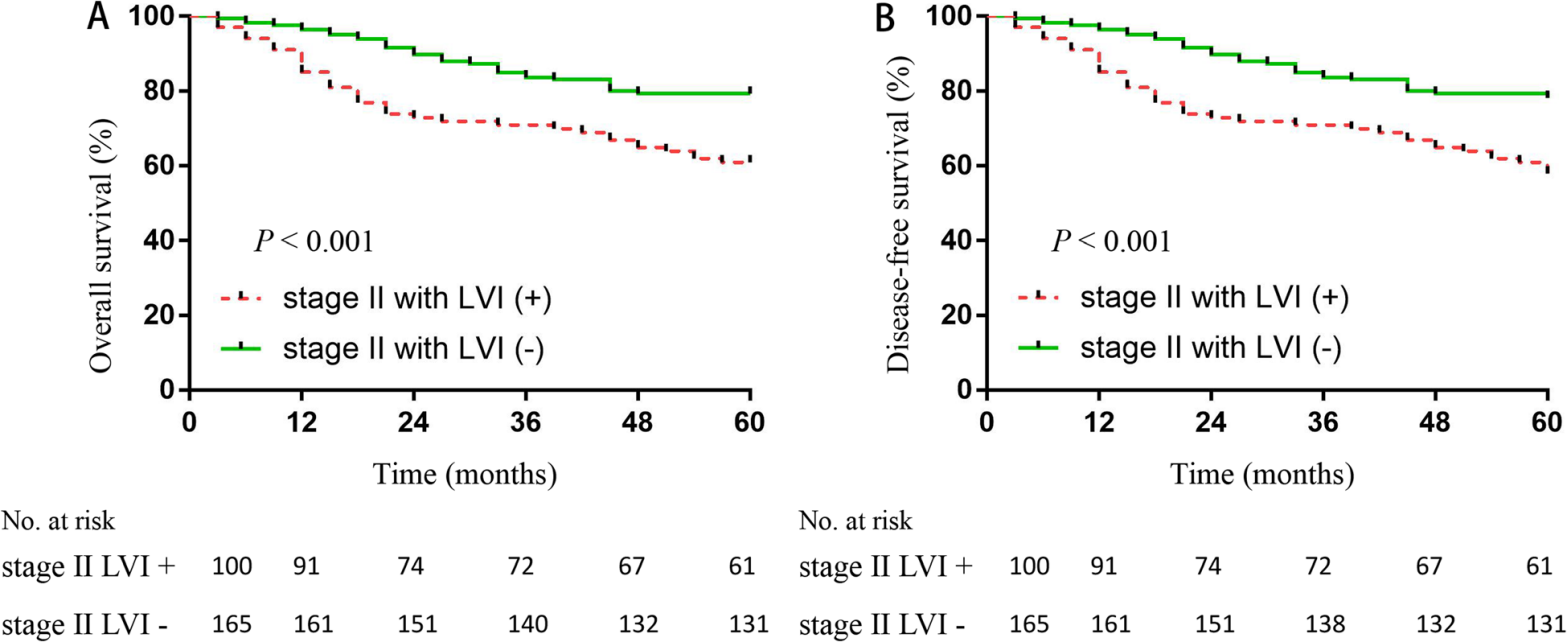
The 5-year overall survival (OS) and disease-free survival (DFS) rates among stage II CRC patients according to lymphovascular invasion (LVI). When dividing the stage II patients into LVI+ and LVI− groups, the 5-year OS rate of the LVI+ group was greater than LVI− (79.4% vs. 61.0%, *P* < 0.001, **a**). The 5-year DFS rate of the LVI− group was greater than the LVI+ group (78.2% vs. 58.0%; *P* < 0.001, **b**)

**Fig. 5:**
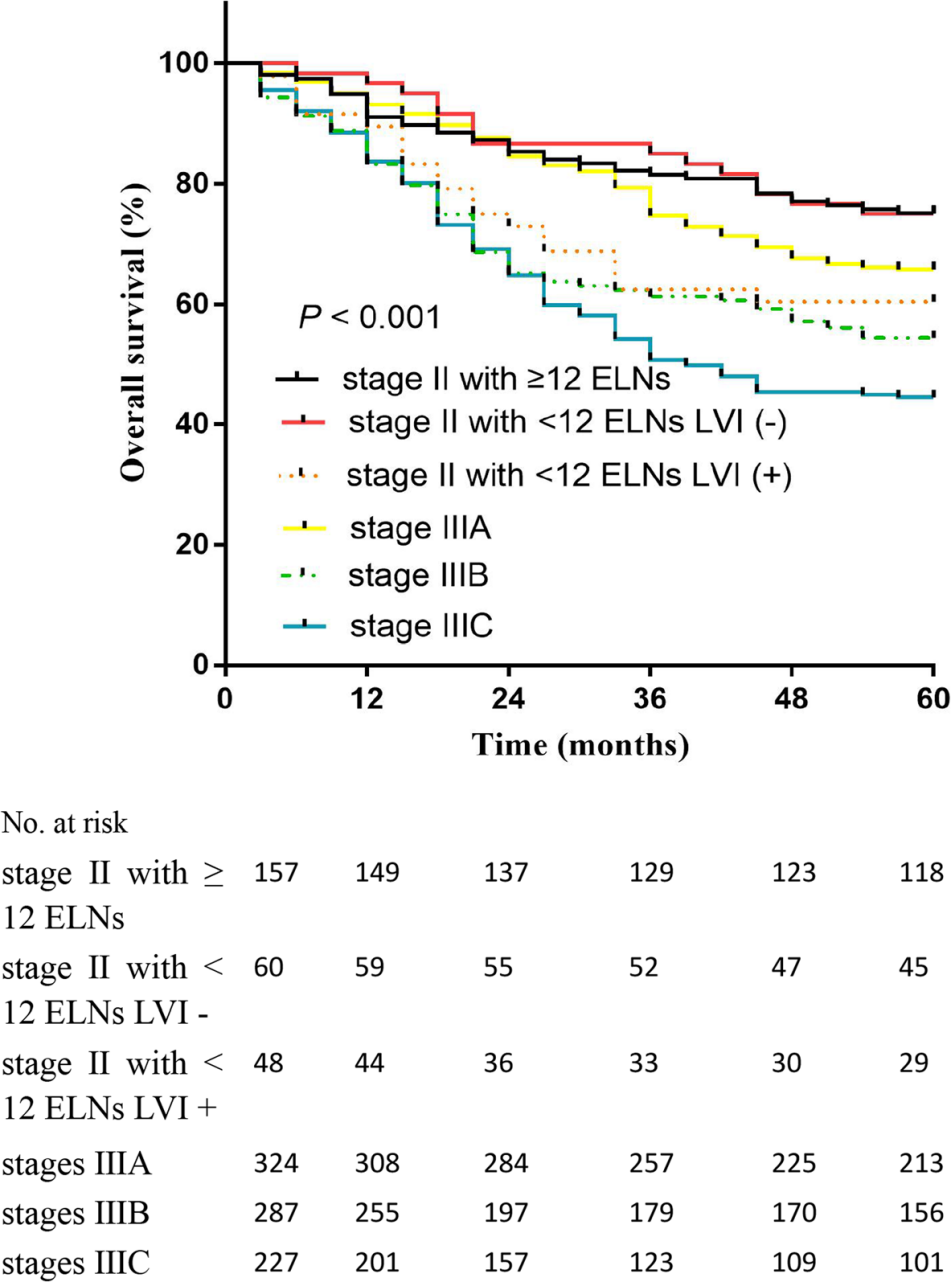
The 5-year overall survival (OS) rates among the different groups. We compared the OS rate among stage II patients with ≥ 12 ELNs; stage II LVI− patients with < 12 ELNs; stage II LVI+ patients with < 12 ELNs; and stages IIIA, IIIB, and IIIC patients. The 5-year OS rate of stage II LVI+ patients with < 12 ELNs was 60.4%, which is significantly lower than stage II LVI− patients with < 12 ELNs and stage II patients with ≥ 12 ELNs, respectively (60.4% vs. 75%, *P* < 0.001; 60.4% vs. 75.2%, *P* < 0.001). No significance existed among stage II LVI+ patients with < 12 ELNs and stages IIIA and IIIB (60.4% vs. 65.7% vs. 54.3 %, *P* = 0.052)

**Fig. 6:**
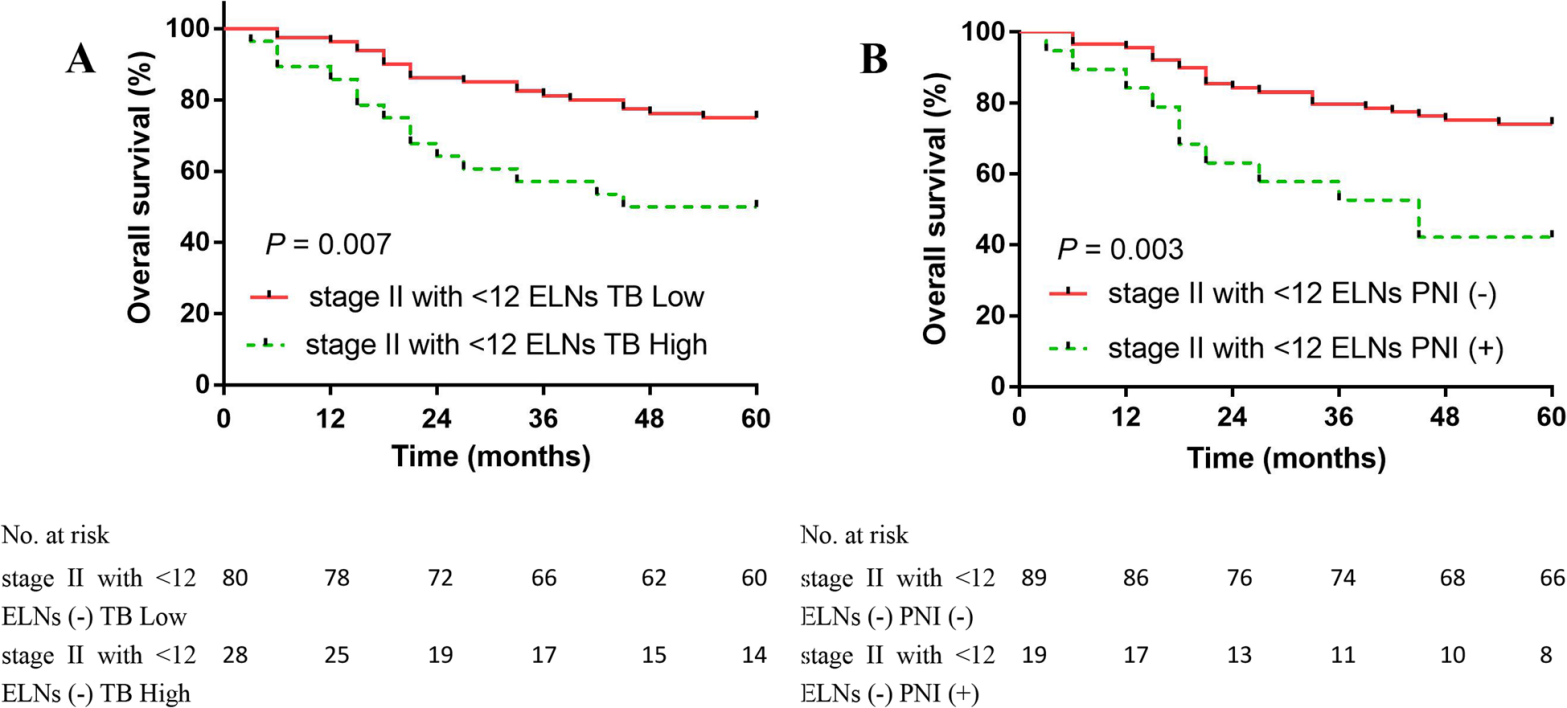
The 5-year OS rate of the TB low group was greater than the TB high group (75.0% vs. 50.0%, *P* = 0.007, **a**). The 5-year OS rate of the PNI − group was greater than the PNI + group (74.2% vs. 42.1%, *P* = 0.003, **b**)

We found that in all stage II patients, the 5-year OS rate of the LVI− group was greater than the LVI+ group (76.5% vs. 58.6%; *P* < 0.001; Fig. 3a). We divided the 265 stage II patients into ELNs < 12 and ELNs ≥ 12 groups. The 5-year OS rate of the ELNs ≥ 12 group (75.2%) was greater than the ELNs < 12 group (68.5%); however, there was no statistically significant difference (Fig. 3b). The 5-year OS rate of the LVI− group was greater than the LVI+ group (79.4% vs. 61.0%; *P* < 0.001; Fig. 4a). The 5-year DFS rate of the LVI− group was greater than the LVI+ group (78.2% vs. 58.0%; *P* < 0.001; Fig. 4b). We further compared the OS rates among stage II patients with ≥ 12 ELNs; stage II LVI− patients with < 12 ELNs; stage II LVI+ patients with < 12 ELNs; and stages IIIA, IIIB, and IIIC patients (Fig. 5). The 5-year OS rate of stage II LVI+ patients with < 12 ELNs was 60.4%, which is significantly less than stage II LVI− patients with < 12 ELNs and stage II patients with ≥ 12 ELNs, respectively (60.4% vs. 75%, *P* < 0.001; 60.4% vs. 75.2%, *P* < 0.001); the 5-year OS rate of stage II LVI+ patients with < 12 ELNs was even lower than stage IIIA; however, there was no significant differences between the two groups (*P* > 0.05). No significant differences existed between stage II LVI+ patients with < 12 ELNs and stages IIIA and IIIB patients (60.4% vs. 65.7% vs. 54.3 %, *P* = 0.052). The 5-year OS rate of the TB low group was greater than the TB high group (75.0% vs. 50.0%; *P* = 0.007; Fig. 6a). The 5-year OS rate of the PNI − group was greater than the PNI + group (74.2% vs. 42.1%; *P* = 0.003; Fig. 6b).

### Univariate and multivariate analyses for the prognosis of stage II patients with < 12 ELNs

Owing to the specific characteristics of stage II patients with < 12 ELNs, the prognostic factors were further analyzed. Univariate analyses showed that LVI, pT-stage, degree of differentiation, and CEA and CA19-9 levels were significant prognostic factors for stage II patients with < 12 ELNs; further multivariate analysis identified that LVI, pT-stage, degree of differentiation, CEA and CA19-9 levels, PNI, TB, and *KRAS* status were significant prognostic factors for stage II patients with < 12 ELNs (all *P* < 0.05) (Table 3).

**Table 3:** Univariate and multivariate analysis of prognostic factors for stage II patients with <12 ELNs. We analyzed the prognostic factors for those patients. As listed in Table 3, univariate analysis showed that LVI, pT-stage, degree of differentiation, serum CEA and CA19-9 levels, perineural invasion, tumor budding, and *KRAS* status were significant prognostic factors for those patients

Parameters	Patients	5-year OS (%)	Univariate analysis		Multivariate analysis	
	*n* (%)		HR (95% CI)	*P*	HR (95% CI)	*P*
Sex				0.675		
Female	53	67.9				
Male	55	69.1				
Age (years)				0.255		
≤65	54	64.8				
>65	54	72.2				
Tumor site				0.384		
Colon	57	63.2				
Rectum	51	68.6				
Tumor size (cm)				0.078		
≤4	60	61.7				
>4	48	77.1				
Lymphovascular invasion			2.313 (1.897–4.562)	0.016	2.313 (1.897–4.562)	0.033
Positive	48	60.4				
Negative	60	75.0				
T-stage			2.358 (1.767–3.897)	<0.001	2.358 (1.767–3.897)	0.001
T3	59	81.4				
T4	49	53.1				
Differentiation degree			1.879 (1.223-4.563)	0.002	1.879 (1.223-4.563)	0.044
Well	53	77.4				
Moderate	53	62.3				
Poor	2	0				
CEA			3.011 (1.997–4.967)	<0.001	3.011 (1.997–4.967)	<0.001
≤5ng/ml	61	78.7				
>5ng/ml	47	55.3				
CA19-9			1.935 (1.156–3.768)	0.001	1.935 (1.156–3.768)	0.015
≤37U/ml	69	75.4				
>37U/ml	39	56.4				
KRAS status			2.430 (1.238–4.770)	0.010	2.277 (1.115–4.653)	0.013
Wild type	68	76.5				
Mutant type	40	55.0				
PNI			2.848 (1.386–5.852)	0.004	2.837 (1.090–5.385)	0.003
Positive	19	42.1				
Negative	89	74.2				
Tumor budding			2.480 (1.251–4.916)	0.009	2.472 (1.099–5.559)	0.007
Low	80	75.0				
High	28	50.0				

*CRC* Colorectal cancer; *LN* Lymph nodes; *ELNs* Examined lymph nodes; *OS* Overall survival; *PNI* Perineural invasion; *TB* Tumor budding

### Improvement of the 8th TNM staging system

Because of the similarity in 5-year OS rates between stage II LVI+ patients with < 12 ELNs and stages IIIA and IIIB patients, we combined LVI with the 8th TNM staging system. A comparison was made to estimate the prognostic value between the new system and the 8th TNM staging system (Table 4). Stage II LVI+ patients with < 12 ELNs were upgraded to stage III, while stage II LVI− patients with < 12 ELNs remained stage II. The 8th TNM staging system combined with LVI had a higher C-index than the 8th TNM staging system alone (C-index, 0.895 vs. 0.833), which indicates a better prognostic value for CRC patients.

**Table 4:** Comparison of the performance of the 8th edition of the TNM Staging System alone and the 8th edition of the TNM Staging System combined with LVI. We combined the LVI with the 8th TNM staging system. A comparison was made to estimate the prognostic value between the new system and the 8th TNM staging system. As listed in Table 4, the 8th TNM staging system combined with LVI had a higher C-index than the 8th TNM staging system alone (C-index, 0.895 vs. 0.833), which indicates a better prognostic value for CRC patients

Classification	Stage	*n*	5-year OS (%)	C-index	95% CI
8th TNM	I	173	90.2	0.833	0.785–0.889
	II	409	72.5		
	III	838	56.2		
8th TNM+LVI	I	173	90.2	0.895	0.812–0.924
	II	361	74.2		
	III	886	56.9		

*C-index* Harrell’s concordance index; *LVI* Lymphovascular invasion; *OS* Overall survival; *CI* Confidence interval

### Relationship between LVI and ACT in stage II CRC patients

**Fig. 7:**
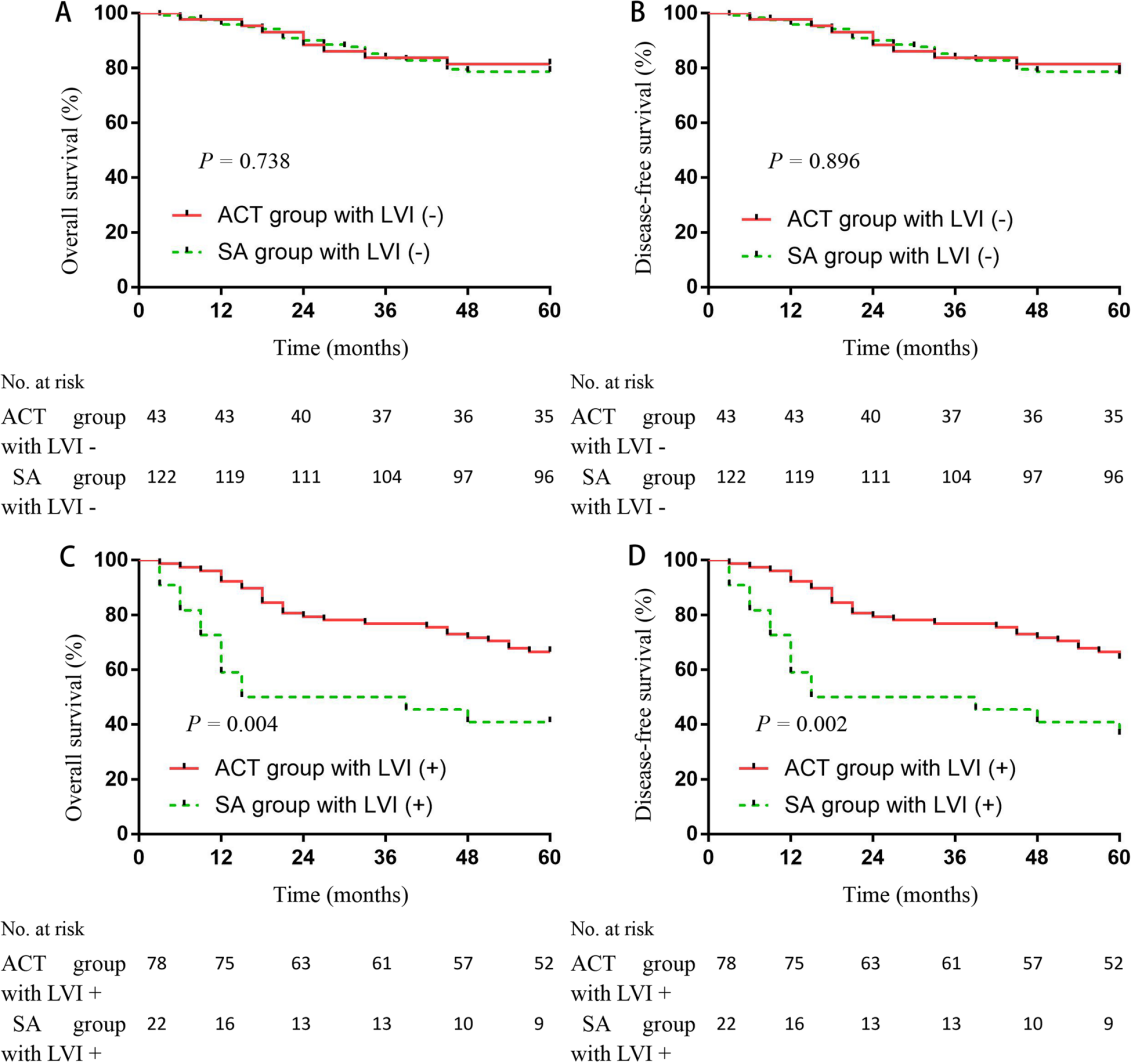
The 5-year overall survival (OS) and disease-free survival (DFS) rates among stage II CRC patients according to adjuvant chemotherapy (ACT). We divided the stage II CRC patients into ACT and surgery alone (SA) groups. In stage II LVI− patients, no significant difference existed in the 5-year OS and DFS rates between the ACT and SA groups (5-year OS, 81.4% vs. 78.7%; *P* = 0.738 (**a**) and 5-year DFS, 79.1% vs. 77.9%, *P* = 0.896; (**b**)). When LVI+ patients were analyzed, the ACT group had significantly higher 5-year OS and DFS rates than the SA group (5-year OS, 66.7% vs. 40.9%, *P* = 0.004, (**c**) and 5-year DFS, 64.1% vs. 36.3%, *P* = 0.002; (**d**))

In addition to analyzing OS, we also analyzed disease-free survival (DFS), especially in stage II CRC patients. The 5-year DFS rate of the LVI− group was greater than the LVI+ group (78.2% vs. 58.0%; *P* < 0.001; Fig. 4b). We further divided the stage II CRC patients into ACT and SA groups. There was no significant difference in the 5-year OS and DFS between stage II CRC patients in the ACT and SA groups (5-year OS, 81.4% vs. 78.7%, *P* = 0.738, Fig. 7a; and 5-year DFS, 79.1% vs. 77.9%, *P* = 0.896, Fig. 7b). When LVI+ patients were analyzed, however, ACT group patients had significantly higher 5-year OS and DFS rates than the SA group (5-year OS, 66.7% vs. 40.9%, *P* = 0.004, Fig. 7c; and 5-year DFS, 64.1% vs. 36.3%, *P* = 0.002, Fig. 7d).

## Discussion

CRC has become a major public health issue worldwide, with 1.4 million new cases and 0.7 million deaths each year [20]. Curative surgery with or without chemotherapy and radiotherapy is the mainstay of treatment for CRC [21]. ACT is recommended for stages III and IV CRC patients [4]. It has been reported that ACT improves OS in stage III patients [22]; however, the benefit of ACT in stage II CRC patients is controversial.

The 8th TNM staging system remains the most important prognostic indicator for CRC patients [8]. For pN stage patients, at least 12 ELNs are recommended to avoid false-negative prognostication; however, it is unavoidable that some cases have < 12 ELNs, which may interfere with the nodal classification and even influence prognosis. LVI has been reported to occur in 10–89.5% of CRC patients [23], which is also considered to increase the risk for micrometastases in localized cancer [24]. Thus, in this study, we focused on stage II LVI+ patients with < 12 ELNs.

As a common histopathologic finding, LVI serves as a prognostic risk factor in many carcinomas [23, 25, 26]. In this study, the LVI+ rate was 34.2% among all CRC patients and 37.7% of stage II CRC patients, which is in agreement with previous studies [20]. Differences in the LVI+ rate might reflect the diagnostic technique used and the number of patients in various studies [27]. In our 100 stage II LVI+ patients, LVI was significantly correlated with pT stage, degree of differentiation, tumor stage, serum CEA and CA19-9 levels, *KRAS* status, TB, and PNI. Similar to our results, Lim et al. [23] reported an association between LVI and more advanced T and N categories, higher pre-CEA levels, and worse tumor grade. Al-Sukhni et al. [28] also concluded that LVI is related to several factors in patients with advanced CRC, including larger size, more advanced T stage, LN involvement, and distant metastasis. Zhong et al. [1] also showed that LVI is significantly associated with an increased CEA level, increased tumor differentiation, and advanced tumor stage. These studies all support our results. Thus, it has been suggested that the presence of LVI should serve as an indicator of extending the resection area [29].

Survival analyses were conducted in this study. The 5-year OS and DFS rates in stage II LVI+ patients were significantly less than LVI− patients. We even found that stage II LVI+ patients with < 12 ELNs had a poor 5-year OS rate that was similar to stage III CRC patients. Multivariate analysis showed that LVI, *KRAS* status, TB, and PNI were significant prognostic factors for stage II CRC patients. Similar to our conclusion, it has been shown that LVI is an independent poor prognostic factor for survival among CRC patients [30]. Huh et al. [31] also reported that N0 stage CRC patients, especially stage II, may benefit most from the presence of LVI because these patients may have a superior response to ACT. The current meta-analysis shows that mutations in the *KRAS* gene appear to be associated with OS in CRC patients [32]. However, another study found that *KRAS* and *BRAF* mutations are independent poor prognostic factors for the OS of stage IV tumors rather than stages I–III tumors [33]. Jang et al. [34] concluded that *KRAS* mutations are significantly associated with high-grade TB; furthermore, tumors with *KRAS* mutations in exons 3 and 4 tended to have LVI and PNI. Marx et al. [35] concluded that higher TB status is related to higher tumor grade and stage, positive lymph nodes, and LVI; similar to our conclusion, it has been shown that TB is an independent poor prognostic factor for survival among CRC patients. Al-Sukhni et al. [28] reported an association between LVI, PNI, and advanced CRC and found that PNI is an independent poor prognostic marker for survival in CRC. Skancke et al. [4] also showed that LVI and PNI have an adverse effect on the survival of patients with stage II colon cancer. When LVI and PNI are present, ACT may have a protective effect.

To explore the benefit from ACT, we focused on the survival of ACT patients with or without LVI. Our results showed that ACT improved the 5-year OS and DFS rates in LVI+ patients. Several studies have reported that ACT is beneficial for stage II CRC patients [36, 37]. Similar conclusions were reported by Skancke et al. [4], who demonstrated that CRC patients with high-risk factors, including LVI, can benefit from ACT. Arakawa et al. [38] reported that a significant prognostic benefit is achieved after ACT for stage IIb/c CRC patients. Lin et al. [39] enrolled 1039 stage II CRC patients and concluded that ACT improves the DFS rate in high-risk stage II CRC patients.

It has been reported that the improvement in OS and DFS rates with ACT did not differ significantly between high- and low-risk stage II CRC patients [22]. Fu et al. [3] suggested that the value of ACT in stage II colon cancer is much less than previously thought; de-escalating chemotherapy for these patients is necessary. Booth et al. [40] reported that ACT is not related to improved survival for stage II CRC patients with high-risk factors. Although there exist some differences in opinions, we still believe ACT is beneficial for LVI+ patients. The current therapy strategies for N0 stage CRC patients do not directly account for LVI.

To further explain the prognostic value of LVI in CRC patients, we combined LVI with the 8th TNM staging system, which had a better predictive efficacy than the 8th TNM staging system alone. Incorporating the negative impact of LVI into the staging system of CRC may predict the prognosis with greater precision and further establish a more reasonable therapeutic strategy for stage II CRC patients.

This study had some limitations. This was a retrospective study from a single center, and the sample size was not sufficiently large, which may have led to selection bias. A multicenter collaborative study with a large sample size may overcome this issue. In addition, we only focused on the phenomenon and the consequences resulting from LVI; thus, it is necessary for us to explore the genetic mechanism underlying LVI, which may provide novel biomarkers and establish new tumor therapeutic strategies for CRC.

## Conclusions

LVI, TB, and PNI are independent prognostic risk factors for stage II CRC patients. Stage II CRC with inadequate ELNs and LVI+ could benefit from adjuvant chemotherapy. The inclusion of LVI can improve the predictive accuracy of the 8th TNM staging system for CRC prognosis. ACT in stage II CRC patients with LVI+ is beneficial for survival.

## Data Availability

The datasets used and/or analyzed during the current study are available from the corresponding author on reasonable request.

## Abbreviations

LVI: Lymphovascular invasion
CRC: Colorectal cancer
ELNs: Examination of lymph nodes
ACT: Adjuvant chemotherapy
PNI: Perineural invasion
TB: Tumor budding
OS: Overall survival
DFS: Disease-free survival
LNs: Lymph nodes
TNM: Tumor-node-metastasis
